# Hip arthroscopy and periacetabular osteotomy generally improve sexual function in patients, but have a risk of iatrogenic pudendal nerve injury that can temporarily worsen sexual function: A systematic review

**DOI:** 10.1002/ksa.12700

**Published:** 2025-05-19

**Authors:** Madeline Hubbard, Darya Pascarel, Prushoth Vivekanantha, Mahmoud Almasri, Shahbaz Malik, Amit Meena, Darren de SA

**Affiliations:** ^1^ Michael G. DeGroote School of Medicine McMaster University Hamilton Ontario Canada; ^2^ Division of Orthopaedic Surgery, Department of Surgery McMaster University Hamilton Ontario Canada; ^3^ Cincinatti Sports Medicine and Orthopaedic Center, Mercy Health Cincinnati Ohio USA; ^4^ Department of Orthopaedic Surgery Worcestershire Acute Hospitals NHS Trust Worcester UK; ^5^ Department of Orthopaedics and Trauma Shalby Hospital Jaipur Jaipur India

**Keywords:** hip arthroscopy, nerve injury, quality of life, sexual function

## Abstract

**Purpose:**

To summarise how orthopaedic hip sports medicine procedures affect patients' sexual function so that surgeons can better counsel their patients on this topic.

**Methods:**

Three databases (MEDLINE, EMBASE and PubMed) were searched on 27 April 2024 with search terms relating to sexual activity and orthopaedic procedures. The authors adhered to the PRISMA and R‐AMSTAR guidelines and Cochrane Handbook for Systematic Reviews of Interventions.

**Results:**

Seventeen studies with a total of 5976 patients (6275 joints) were included in this study. Hip arthroscopies were performed in 5812 patients for a total of 6087 surgeries, and 164 patients received 188 osteotomies. Nine of 17 studies reported iatrogenic nerve injury (103/1854; 5.6%), mainly of the pudendal nerve (64/103, 62.1%). All of male, female, and combined male and female sexual function tended to be compromised prior to hip sports medicine surgery and improved after surgery as per International Index of Erectile Function (IIEF) (*p* = 0.009) and Female Sexual Function Index (FSFI) (*p* < 0.001) scores. Improvements after surgery were largely due to decreased hip pain and stiffness during sexual activity. Return to sexual activity was reported to be 29.2 ± 20.1 days after hip arthroscopy. Only three studies discussed preoperative counselling on sexual activity.

**Conclusion:**

Hip sports medicine surgeries can improve sexual function for patients; however, they have a risk of pudendal nerve damage that can temporarily interfere with sexual function. Surgeons should counsel their patients on the risks and benefits of hip sports orthopaedic surgeries to sexual function.

**Level of Evidence:**

Level IV.

**Abbreviations**
FSFI: female sexual function index
IIEF: international index of erectile function
MINORS: Methodological Index for Non‐randomised Studies
PRISMA: preferred reporting items for systematic reviews and meta‐analyses
R‐AMSTAR: revised assessment of multiple systematic reviews
SAQ: sexual activity questionnaire

## INTRODUCTION

Sexual health is a vital part of patients' holistic well‐being involving physical, emotional, mental and social well‐being related to an individual's sexuality [12]. Some validated tools used to measure sexual health are the Sexual Activity Questionnaire (SAQ), the Female Sexual Function Index (FSFI) for female patients, the International Index of Erectile Function (IIEF) and the abridged IIEF‐5 for male patients [37, 38, 39, 44]. Unfortunately, stigma around sexual health can lead physicians to avoid discussing this topic [4]. Strategies to reduce stigma include providing information and skill‐building opportunities for healthcare providers to discuss sexual health [4].

Sexual function is rarely discussed in many orthopaedic patient populations. One study showed that only half of participating surgeons believed that providing information about postoperative sexual activity and function restrictions was necessary after total knee arthroplasty or total hip arthroplasty [14]. Another survey demonstrated that 80% of responding surgeons rarely or never discuss sexual activity with their patients who have had hip arthroplasty [11]. Only 17% of patients received information about returning to sexual activity after total hip arthroplasty; however, 55% of patients would have liked to receive more information on this subject [48].

Sexual health after orthopaedic surgeries is gaining more recognition as an important component of health, as evidenced by previous reviews investigating sexual function after total hip arthroplasty, total knee arthroplasty and spinal cord injury and surgeries [1, 14, 17, 20, 31]. Despite this, there is currently no comprehensive systematic review on postoperative sexual function and activity after hip sports medicine procedures. Hip pathologies, such as acetabular labral tears, have been shown to impair sexual function [36]. Investigating and reporting on sexual function after hip surgeries, including hip arthroscopy and periacetabular osteotomy, may fill a gap in the literature that provides orthopaedic surgeons evidence‐based insights on counselling around sexual health and function in the perioperative setting. Therefore, the primary objective of the current review was to report on patient‐specific outcomes in sexual function and activity after orthopaedic hip sports medicine procedures. It is hypothesised that sexual function improves after orthopaedic hip sports medicine procedures due to improved joint pain and motion.

## MATERIALS AND METHODS

The Preferred Reporting Items for Systematic Reviews and Meta‐Analyses (PRISMA) and Revised Assessment of Multiple Systematic Reviews (R‐AMSTAR) guidelines for coordinating and reporting systematic reviews were followed throughout the development of this manuscript [23, 25].

### Search criteria

A search was completed of three online databases (MEDLINE, EMBASE and PubMed) including literature published on or before 27 April 2024. Search terms were developed that focused on sexual activity and orthopaedic procedures. Studies were selected if they met the following criteria: (1) studies including patients who underwent hip sports medicine procedures (hip arthroscopy, acetabular osteotomy) [47], (2) studies reporting on sexual function or activity after these surgeries; (3) non‐laboratory human studies; and (4) studies in the English language. Exclusion criteria included: (1) level of evidence V; (2) textbook chapters; (3) conference abstracts; (4) biomechanical studies; (5) cadaveric or animal studies; and (6) studies involving less than five patients. The references of included studies and additional review papers were manually searched for additional eligible studies.

### Screening

Two authors (MH and DP) completed title and abstract screening. If a conflict arose that could not be resolved by the two authors, then a third author (PV) made the final decision. Full‐text screening and conflict resolution was completed in a similar manner.

### Assessment of agreement

The kappa (*κ*) statistic for screening was used to assess the inter‐reviewer agreement. Inter‐reviewer agreement was classified a priori as follows: almost perfect agreement was *κ* of 0.91–0.99; considerable agreement was *κ* of 0.71–0.90; high agreement was *κ* of 0.61–0.70; moderate agreement was *κ* of 0.41–0.60; fair agreement was *κ* of 0.21–0.40; and no agreement was *κ* of 0.20 or less [28].

### Quality assessment

The Methodological Index for Non‐Randomised Studies (MINORS) criteria were used for methodological quality assessment [41]. Based on the MINORS criteria, non‐comparatives could get a maximum score of 16. Based on a previous systematic review, the classification was a priori for non‐comparative studies: 0–4 indicated very low quality evidence, 5–7 indicated low quality evidence, 8–12 indicated fair quality evidence and scores ≥ 13 indicated high quality evidence [10]. Similarly, a priori classification for comparative studies, which could have a maximum score of 24, was: 0–6 indicated very low quality evidence, 7–10 indicated low quality evidence, 11–15 indicated fair quality evidence, 16–20 indicated good quality evidence and score ≥ 20 indicated high quality evidence [10].

### Data abstraction

Google Sheets (Google LLC, Mountain View, CA, USA) was used for data abstraction. Demographic data extracted included the number of patients and joints, mean follow up time, age, and body‐mass index (BMI). Data regarding the injury or pathology (e.g., femoroacetabular impingement, labral tear, hip dysplasia, etc.), surgical procedure and rehabilitation details were also extracted. Sexual demographics were recorded including if the patient was the receptive or penetrative partner or both. Any scores or assessment tools used to describe sexual activity or function were extracted (e.g., SAQ, FSFI and IIEF‐5) pre‐injury (if applicable), pre‐surgical and post‐surgical. Additionally, subjective measurements of sexual function were extracted (e.g., pain, satisfaction and position preference), as well as time to return to sexual activity postoperatively. This was extracted in the form of how it was reported.

### Outcome reporting

Results were presented in a narrative format due to the diversity in outcomes reported. Objective measurements were presented with means, ranges, percentages and standard deviation, which were calculated using Google Sheets (Google LLC, Mountain View, Ca, USA). As well, *p*‐values were recorded if they were included if they were reported in the study. *p*‐Values below 0.05 were considered as significant.

## RESULTS

### Literature search

**Figure 1:**
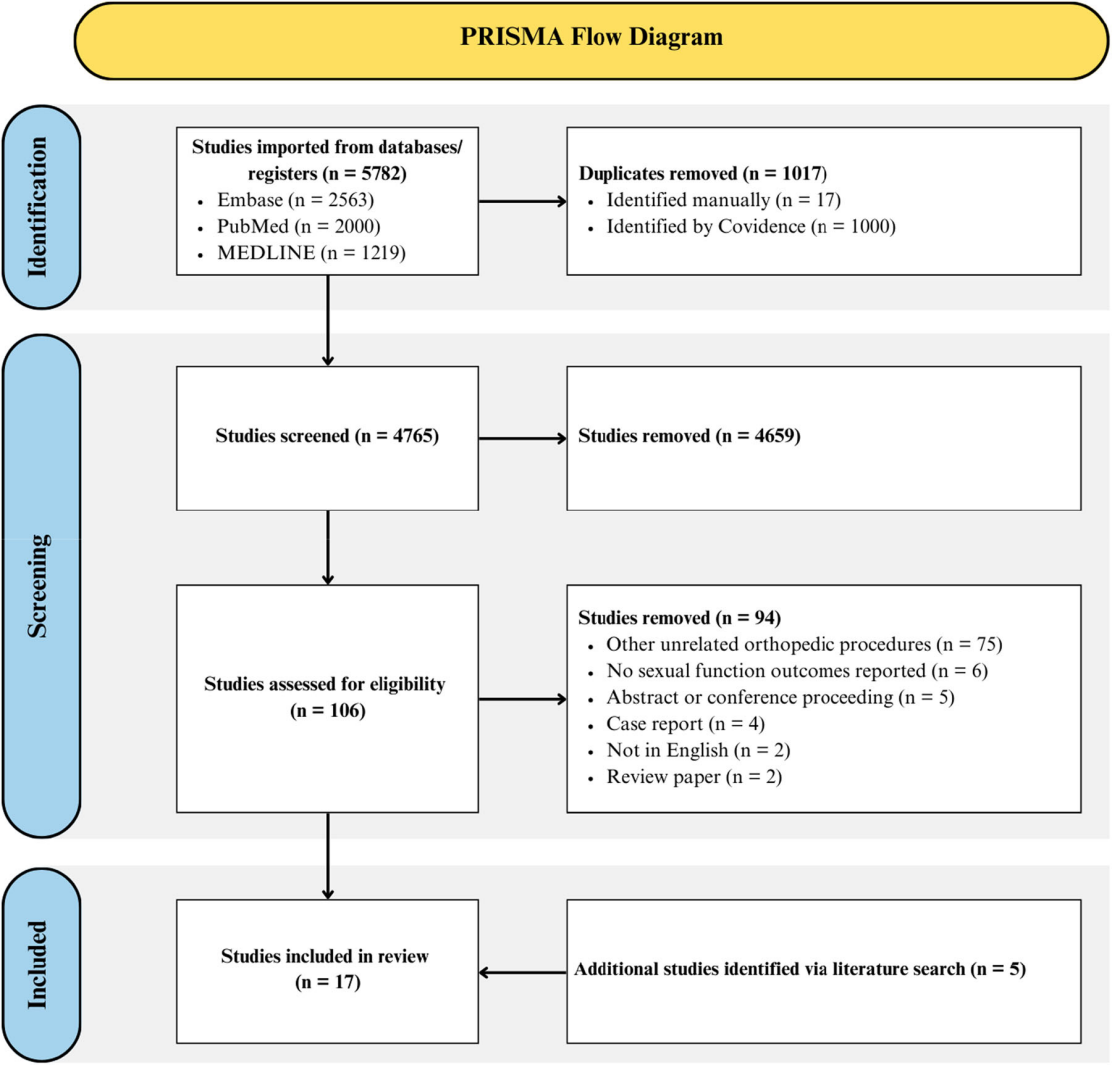
Preferred Reporting Items for Systematic Reviews and Meta‐analyses (PRISMA) flow diagram representing a systematic review outlining sexual function after hip sports medicine orthopaedic procedures.

A PRISMA flow diagram is presented in Figure 1 depicting literature search and screening results. Overall, 17 studies were identified as meeting inclusion and exclusion criteria for this review. There was considerable agreement during both the title and abstract screening stage (*κ* = 0.734, 95%CI = 0.670–0.798) and the full‐text screening stage (*κ* = 0.767, 95%CI = 0.589–0.945).

### Study quality

Most studies included in this review were case series or cohort studies (level of evidence IV) (Table 1). The mean MINORS score for non‐comparative studies was 10.4/16 with all studies being considered fair quality. One comparative study was considered good quality with a score of 19/24 (Table 1).

**Table 1:** Demographic data.

Author (year)	Study design (level of evidence)	Number of patients	Number of joints	Mean (SD) [range] age (years)	Female, *n* (%)	Mean (SD) [range] follow‐up time (months)	Loss to follow‐up (%)	MINORS score
Alkan et al. [2]	Retrospective cohort analysis (IV)	96	96	35.3 (8.3) [18–51]	44	32.3 (17.5) [12–82]	0	11/16
Bozic et al. [5]	Retrospective database analysis (IV)	1014	1014	FAI: 38.0 (13.8) Labral repair: 37.7 (12.6)	60	NR	N/A	9/16
Byrd et al. [6]	Case control study (III)	130 (Study: 108, Control: 122)	144 (Study: 122, Control: 122)	Study: 15.9 (1.1) [12–17] Control: 36.8 (8.8) [18–50]	Study: 55 Control: 42	30 [12–60]	1.6 control patients	19/24
Carreira et al. [7]	Prospective case series (IV)	45	45	40.0 [16–69]	64	12	0	12/16
Coady et al. [9]	Prospective case series (IV)	26	26	42 [23–74]	100	[36–58]	0	12/16
Holderread et al. [16]	Cross‐sectional analysis of anonymous online discussion platforms (IV)	23	23	Not reported	50 (8/16)	N/A	N/A	10/16
Jean et al. [18]	Convenience sample from a multi‐centred RCT (FIRST) and prospective cohort study (EPIC) (IV)	324 (FIRST: 214, EPIC: 110)	324	FIRST: 35.9 (8.9) EPIC 33.1 (8.9)	FIRST: 42 EPIC: 33	6 Weeks: 138 patients 12 months: 143 patients	FIRST: 3 EPIC: 4	11/16
Kern et al. [21]	Prospective cohort study (IV)	100	100	30.11 (11.84) [13–62]	63	Until symptoms resolved (≤9 mo.)	0	12/16
Klit et al. [22]	Retrospective cohort, cross‐sectional review (IV)	55	68	Surgery: 31 [14–56]; Follow up: 41 [24–67]	77	10 [9–12]	5.5	11/16
Lee et al. [24]	Retrospective case series (IV)	131	131	35.2 (11.6)	57	21.0 (5.4)	0	10/16
Masui et al. [26]	Retrospective case series (IV)	21	28	Surgery: 25.7 [18–35] First delivery: 30.7 [23–40]	100	92.4 [24–144]	0	11/16
Park et al. [34]	Retrospective case series (IV)	197	200	44.64 [19–70]	51	28.2 [19–42]	0	11/16
Raut et al. [35]	Retrospective case series (IV)	92	92	[16–61]	100	43 [23–85]	0	10/16
Rynecki et al. [40]	Retrospective case series (IV)	61	61	34 (9)	56	24 (12)	0	10/16
Smith et al. [42]	Retrospective case series (IV)	3438 patients	3691	Median [IQR] post‐op 6 mo.: 36.2 [29–44] 12 mo.: 36.4 [29–44]	62	NR	0	10/16
Valenzuela et al. [46]	Retrospective case series (IV)	88	92	32 [17–56]	100	3.8 [1–8]	0	9/16
Welton et al. [49]	Case series (IV)	35	40	32.3 [18–50]	60	[7–12 days]	0	8/16

Abbreviations: FAI, femoroacetabular impingement; IQR, interquartile range; MINORS, methodological index for non‐randomised studies; NR, not reported; SD, standard deviation.

### Study characteristics

The 17 studies in this review consisted of 5976 patients (6275 joints) [2, 5, 6, 7, 9, 16, 18, 21, 22, 24, 26, 34, 35, 40, 42, 46, 49]. The mean age of included patients was 34.5 years (range: 25.7–44.6 years). The mean BMI of included patients was 24.9 kg/m² (range: 24.3–25.4 kg/m²) amongst three studies reporting values [2, 7, 21]. Females comprised 66.5% (range: 38.9–100%) of included patients. The mean follow‐up time was 29.7 months (range: 3.8–92.4 months) amongst 10 studies reporting values [2, 6, 7, 22, 24, 26, 34, 35, 40, 46]. Full demographic details can be found in Table 1.

### Hip surgical indications and characteristics

Hip arthroscopy was the most common procedure performed, being reported in 14 papers for a total of 5812 patients receiving 6087 procedures (Table 2) [2, 5, 6, 7, 9, 16, 18, 21, 24, 34, 35, 40, 42, 49]. The remaining three papers described use of acetabular osteotomy in the treatment of hip dysplasia for a total of 164 patients receiving 188 procedures [22, 26, 46]. Seven (50%) papers described use of a perineal post during hip arthroscopy [2, 7, 16, 18, 21, 34, 40], while one (7.1%) paper described postless surgery [49]. Eight (57.1%) papers described use of traction during hip arthroscopy [2, 7, 9, 16, 18, 21, 34, 49], with an average duration of 77.9 minutes (range: 42.0–81.6 min) amongst four studies reporting values (Table 2).

**Table 2:** Pathology and surgery details.

Author (year)	Pathology	Surgical procedure	Iatrogenic nerve injury
Alkan et al. [2]	Femoroacetabular impingement	Hip arthroscopy (Post +, Traction +)	30.2% (pudendal)
Bozic et al. [5]	Femoroacetabular impingement and/or labral tear	Hip arthroscopy (Post NR, Traction NR)	0.3% (pudendal) 0.8% (lateral femoral cutaneous) 0.1% (sciatic) 0.5% (superficial peroneal) 0.1% (peroneal) 0.1% (long thoracic nerve)
Byrd et al. [6]	Femoroacetabular impingement	Hip arthroscopy (Post NR, Traction NR)	2.3% (perineal) 0.7% (lateral femoral cutaneous)
Carreira et al. [7]	Femoroacetabular impingement	Hip arthroscopy (Post +, Traction +)	2.2% (pudendal) 13.3% (distal anterolateral thigh) 15.6% (other)
Coady et al. [9]	Femoroacetabular impingement	Hip arthroscopy (Post NR, Traction +)	NR
Holderread et al. [16]	Femoroacetabular impingement	Hip arthroscopy (Post +, Traction +)	65.0% (pudendal)
Jean et al. [18]	Femoroacetabular impingement	218 patients arthroscopic osteochondroplasty. 106 patients arthroscopic lavage with or without labral repair. (Post +, Traction +)	NR
Kern et al. [21]	All pathology requiring hip arthroscopy excluding extra‐articular pathologies	Hip arthroscopy (Post +, Traction +)	9% (Pudendal) 2% (Lateral femoral cutaneous) 1% (Sciatic) 1% (Superficial peroneal nerves)
Klit et al. [22]	Acetabular dysplasia of the hip	Periacetabular osteotomy	NR
Lee et al. [24]	Femoroacetabular impingement	Hip arthroscopy (Post NR, Traction NR)	NR
Masui et al. [26]	Developmental dysplasia of hip: Osteoarthritis of hip secondary to hip dysplasia or Legg‐Perthes disease	Eccentric rotational acetabular osteotomy	NR
Park et al. [34]	Femoroacetabular impingement	Hip arthroscopy (Post +, Traction +)	2% (pudendal) 1% (lateral femoral cutaneous)
Raut et al. [35]	Hip acetabular labral tear	Hip arthroscopy (lateral position, three‐portal technique) (Post NR, Traction NR)	NR
Rynecki et al. [40]	Femoroacetabular impingement	Hip arthroscopy (labral debridement/repair, femoral head/neck osteochondroplasty, and acetabuloplasty) (Post +, Traction NR)	NR
Smith et al. [42]	Femoroacetabular impingement	Hip arthroscopy (Post NR, Traction NR)	NR
Valenzuela et al. [46]	Acetabular dysplasia	Acetabular osteotomy	4.5% (Peroneal nerve)
Welton et al. [49]	Not mentioned	Hip arthroscopy (Post ‐, Traction +)	0%

Abbreviations: NR, not reported; Post, perineal post.

### Iatrogenic pudendal nerve injury after hip surgeries

Eight papers described the incidence of iatrogenic nerve injury following hip arthroscopy and one reported on nerve injury after osteotomy (Table 2) [2, 5, 6, 7, 16, 21, 34, 46, 49]. The mean occurrence of iatrogenic nerve injury ranged between 0% and 65%. One study reported no iatrogenic nerve injury with postless hip arthroscopy [49]. There were 103 reports of nerve injuries out of 1854 patients, giving an overall incidence of 5.6%. The pudendal nerve was implicated in 62.1% (64/103) of iatrogenic nerve injuries [2, 5, 6, 7, 16, 21, 34]. Pudendal nerve injury was temporary in 82.8% (53/64) of patients, taking between 2 weeks to 9 months to resolve in most cases [2, 6, 7, 16, 21, 34]. In 12.5% of patients with pudendal nerve injury, the injury was considered to be permanent [2, 16].

### Male sexual function after hip surgeries

Two studies reported improvements in male sexual function after hip arthroscopy from preoperative status, finding significant improvements in IIEF scores and SAQ scores (Table 3) [2, 42]. Two other studies reported no significant improvements when measuring using a Likert scale after hip osteotomy and IIEF scores after hip arthroscopy [18, 22]. One study found that 6.3% of male patients experienced erectile dysfunction along with decreased sensation of light touch in the perineal area after a hip arthroscopy [7].

**Table 3:** Sexual function after hip orthopaedic procedures.

Author (year)	Patient sex	Score used	Pre‐surgical mean (SD) [range]	Post‐surgical mean (SD) [range]	Pre‐surgical vs. post‐surgical
Alkan et al. [2]	Male	IIEF‐5 (max 30)	20.3 (6.2) [3.0–25.0]	21.9 (3.9) [13.0–25.0]	Sig. different (*p* = 0.009)
	Female	FSFI (max 36)	21.6 (6.7) [6.7–27.5]	23.0 (6.2) [6.7–28.4]	Sig. different (*p* < 0.001)
Jean et al. [18]	Male	IIEF	NR	Mean difference [95%CI]: − 6 w: 0.57 [−1.22, 2.37] − 12 mo:1.58 [−0.32, 3.47]	Not different − 6 w (*p* = 0.531) − 12 mo (*p* = 0.103)
	Female	FSFI	NR	Mean difference [95%CI]: − 6w: 1.18 [−2.09, 4.45] − 12 m: ‐2.12 [−4.52, 0.28]	Not different − 6w (*p* = 0.480) − 12 m (*p* = 0.083)
Klit et al. [22]	Male	Likert scale of ability in sex life (max 6)	5.5 [3–6]	6.0 [5–6]	Not different (*p* = 0.102)
	Female	Likert scale of ability in sex life (max 6)	4.0 [1–6]	5.0 [2–6]	Sig. different (*p* = 0.008)
Smith et al. [42]	Male	SAQ	Median [IQR]: 50.0 [25–84]	Median [IQR]: − 6 mo: 80.0 [45–91] − 12 mo: 80.0 [41–98]	Sig. different (*p* < 0.001)
	Female	SAQ	Median [IQR]: 30.0 [14–50]	Median [IQR]: − 6 mo: 60.0 [28–86] − 12 mo: 62.0 [29–90]	Sig. different (*p* < 0.001)
	Combined	SAQ	Median [IQR]: 35.0 [18–66]	Median [IQR]: − 6 mo: 70 [30–90] − 12 mo: 70 [31–91]	Sig. different (*p* < 0.001)
Lee et al. [24]	Combined	Likert scale of sexual function (23 items)	66% sexual difficulties	10.8% sexual difficulties	Sexual activity frequency: − 48.5% no change − 32.3% increase − 16.9% decrease − 2.3% not resumed

Abbreviations: CI, confidence interval; Combined, both male and female patients; FSFI, female sexual function index; IIEF, international index of erectile function; IQR, interquartile range; NR, not reported; SAQ, Sexual Activity Questionnaire; SD, standard deviation; Sig., significantly (*p* < 0.05).

### Female sexual function after hip surgeries

Three studies reported improvements in female sexual function after either a hip arthroscopy or acetabular osteotomy using FSFI scores, a Likert scale or SAQ scores (Table 3) [2, 22, 42].

### Combined male and female sexual function after hip surgeries

Three studies reported improvements in sexual function after hip arthroscopy using SAQ scores, a Likert scale, and FSFI and IIEF scores (Table 3) [2, 24, 42]. Sexual dysfunction was reported in 66% of patients before surgery and only 10.8% of patients after hip arthroscopy [24]. Using FSFI and IIEF scores, 31.1% of patients improved after hip arthroscopy and 52.2% did not improve [2]. The patients who did not improve tended to be younger, have a lower BMI, have had a longer traction time during surgery, were more likely to smoke and were more likely to have had pudendal nerve symptoms post‐operatively [2]. After a hip arthroscopy without a perineal post, no patients reported sexual dysfunction within the follow‐up period of 7–10 days after surgery [49].

### Subjective perceptions of sexual function after hip surgeries

Before surgery, pain, hip stiffness, and loss of interest were all contributors to sexual difficulty (Table 4) [9, 24, 26, 35, 40, 46]. Compared to penetrative partners, receptive partners tended to have more pain and stiffness hindering sexual relations before surgery [40]. In patients with vulvodynia and femoroacetabular impingement, 23.1% had improvements to their symptoms after a hip arthroscopy [9]. These patients tended to be younger and have had a shorter duration of vulvodynia preoperatively [9].

**Table 4:** Subjective sexual function after hip orthopaedic procedures.

Author (year)	Pre‐surgical subjective	Post‐surgical subjective	Time to resume sexual activity	Sexual positions
Coady et al. [9]	Vulvodynia mild (15%), moderate (38%) or severe (46%).	Vulvodynia mild (42%), moderate (23%) or severe (31%). Improvement in 23.1% of patients: tended to be younger (22–29 years) and had vulvodynia or clitorodynia less than 4 years.	NR	NR
Lee et al. [24]	Sexual difficulty due to pain (77.9%), stiffness (47.1%), loss of interest (21.4%).	Sexual activity currently enjoyable (74.8%). Unhappiness in relationship due to sexual difficulties from hip pain (13.7%). Mean (SD) days to return to sexual activity with minimal pain: 48.8 (40.6).	Mean (SD) days: 29.2 (20.1). − Female: 34.8 (23.2) vs. Male: 21.0 (10.7) *p* < 0.0001. − Younger 26.3 (21.7) vs. older 35.7 (13.5) *p* = 0.017.	29.0% of patients changed their sexual positions due to hip pain after surgery: Female: 82.3%, Male: 17.7%, *p* < 0.0001.
Masui et al. [26]	NR	68.4% improved sexual satisfaction: less pain (41.2%), less fear of pain (11.8%), less fear of hip dislocation (17.6%). 21.1% decreased sexual satisfaction: decreased range of motion (17.6%).	NR	NR
Raut et al. [35]	Pain during sex (94%)	Pain improved (89%)	NR	NR
Rynecki et al. [40]	Sexual difficulty due to hip pain (penetrative: 18%, receptive: 32%), hip stiffness (penetrative: 6%, receptive: 32%).	Persistent hip pain at 6 weeks (penetrative: 11%, receptive: 13%). Pain‐free intercourse (penetrative: 52%, receptive: 9%).	5% did not return	Receptive partners pre‐surgical pain in positions with a greater degree of hip flexion and abduction, which improved post‐surgically. No change in penetrative group pain.
			Median [range] − Receptive: 6 w [4–14] − Penetrative: 6 w [2–14]	
Valenzuela et al. [46]	NR	80% of patients: no residual pain or mild pain. 5% of patients: very severe hip pain. Change in frequency (40.4%) due to less pain (39.5%), more pain (18.4%), improved ROM (28.9%), decreased ROM (34.2%). Change in sexual satisfaction (25.5%) due to less pain (54.2%), more pain (8.3%), improved ROM (42.7%), decreased ROM (25.0%).	NR	46% had a change in positions due to less pain (38.6%), more pain (31.8%), improved ROM (22.7%), decreased ROM (45.5%).

Abbreviations: IQR, interquartile range; ROM, range of motion; SD, standard deviation.

Hip pain during sexual activity largely improved for patients who received a hip arthroscopy (Table 4). At an average of 1.75 years after surgery, 74.8% of patients reported that sexual activity was currently enjoyable [24]. However, 13.7% of patients reported unhappiness in their relationship owing to sexual difficulties associated with hip pain [24]. Hip pain was more likely to persist at 6 weeks post‐surgery for receptive partners compared to penetrative partners [40]. Sexual activity and satisfaction also tended to improve for patients who received a hip osteotomy, due to less pain, less fear of pain, less fear of their hip dislocating and improved range of motion (Table 4) [26, 46]. However, some patients experienced more pain or decreased range of motion that interfered with sexual activity after their hip surgery [26, 46].

### Time to resumption of sexual activity after hip surgeries

Patients returned to sexual activity an average of 29.2 ± 20.1 days, or a median of 6 weeks (range 2–14 weeks) after surgery (Table 4) [24, 40]. A quicker return to sexual activity was found in male patients and younger patients [24]. Throughout an average follow up time of 2 years, 5% of patients did not return to sexual activity [40].

### Positions for sexual activity after hip surgeries

After an acetabular osteotomy, 46% of patients had a change in their positions with sexual activities (Table 4) [46]. This was due to less pain in 38.6% of patients, more pain in 31.8% of patients, decreased range of motion in 45.5% of patients and improved range of motion in 22.7% of patients [46]. Prior to a hip arthroscopy for femoroacetabular impingement, receptive partners reported avoidance of positions that involved a greater degree of hip flexion and abduction [40]. After surgery, there was an improvement in pain for receptive partners in positions of significant hip flexion and abduction [40]. There were no changes to pain in any positions from prior to after surgery for penetrative partners [40]. In another study, 29.0% of patients changed their sexual positions after hip arthroscopy due to hip pain, with significantly more females requiring an adjustment to their sexual positions [24].

### Preoperative counselling on sexual activity

Three studies reported on preoperative counselling on either return to sexual activity or potential sexual complications after hip arthroscopy for femoroacetabular impingement [16, 24, 40]. In one study, patients were counselled that they did not have any postoperative limitations on sexual activity, and they could return to sexual activity when they were comfortable doing so [40]. In another study, preoperative counselling was reported by 8.7% of patients regarding the risk of pudendal nerve injury and 4.3% of patients regarding the severity of pudendal nerve damage [16]. After surgery, 30.4% of patients reported receiving information about pudendal injury [16]. In a final study, 28.1% of patients reported being counselled on sexual activity before or after surgery [24]. When asked about how they would prefer to receive information about sexual activity, most patients preferred either a discussion with their surgeon (77.4%) or a printed information source such as a booklet or handout (67.4%) [24].

## DISCUSSION

The most important finding of the current review was that hip sports‐related orthopaedic surgeries tend to improve sexual function for patients. The current review was composed of studies that assessed sexual function after hip arthroscopy for femoroacetabular impingement or acetabular osteotomy for hip dysplasia. In both contexts, patients had more sexual difficulties pre‐operatively than they did post‐operatively. This held true for analyses of male patients alone, female patients alone and combined male and female patients. The improvements in sexual activity tended to be due to decreased pain and stiffness during sexual activity. For some patients, improvements in sexual activity after hip sports‐related orthopaedic procedures could be one of several reasons to pursue surgical intervention.

Sexual function is an important aspect of health that is often ignored or avoided largely due to stigma [4]. Although sexual function is an integral part of holistic wellness, most patients in the current review who received hip sports‐related orthopaedic procedures reported that they did not receive counselling about the potential of pudendal nerve damage with surgery or on safe return to sexual activity after surgery [16, 24]. For orthopaedic surgeons who perform total hip and knee arthroplasty, most surgeons report that they almost never address sexual function with their patients, especially for elderly patients [11, 15, 48]. The cited reasons are that surgeons were not aware of the need to discuss sexual function after total hip arthroplasty, or that patients did not ask about this information [15]. Although the age of the patient population undergoing total hip or knee arthroplasty differs from those included in the current review, there is a consistent trend that patients receiving orthopaedic procedures do not receive adequate preoperative counselling about the implications of their procedure on sexual health. For patients undergoing hip arthroscopy, most patients would prefer to discuss sexual activity with their surgeon, or to receive information in a printed resource [24].

In a survey in patients who underwent reconstructive hip surgery, including total hip arthroplasty, osteotomy, and internal fixation for femur fractures, 57% of patients waited at least 4 months after surgery before resuming sexual activity [29]. When asked why they waited as long as they did to resume sexual activity, most patients responded that they did not receive information from any healthcare provider regarding when it was safe to return to sexual activity [29]. In fact, 83% of patients reported feeling inadequately informed about the effect of their hip surgery on their sexual quality of life, when it was safe to return to sexual activity, and which positions were safe for sexual activity [29]. After total hip arthroplasty, positions involving extreme hip flexion are discouraged as they increase prosthetic impingement and potentially dislocation risk [8]. While dislocation is not a concern after hip arthroscopy, future research is similarly encouraged to identify positional restrictions for sexual activity after labral procedures and/or osteochondroplasty to enhance transparency and communication with patients.

In the current review, two studies reported on the time to return to sexual activity after a hip arthroscopic surgery [24, 40]. According to these studies, male or penetrative partners should wait at least 2–3 weeks to return to sexual activity. In contrast, female or receptive partners should wait at least 4–5 weeks. Previously, female sex was found to possibly be a predictor of more negative outcomes after hip arthroscopy [27], and male patients have been found to have a higher return to sport rate than female patients [45]. Additionally, female patients tended to have lower preoperative sexual function scores than male patients [22, 42]. These reasons could explain why time to return to sexual activity is slightly increased in female or receptive partners. For all patients regardless of sex, the median time to return to sexual activity was 6 weeks [40]. Therefore, a more cautious time frame to counsel patients to return to sexual activity after hip arthroscopy would be 6 weeks, or when sexual activity can be completed without pain. Some patients continue to experience sexual difficulties following hip procedures related to pain or decreased range of motion however, there is a lack of research showing the role of rehabilitation protocols in improving these outcomes [26, 46]. Future research should focus on the role of pain management and physiotherapy.

In addition to differences in return to sexual activity, biological sex influences the positions that are comfortable for patients after surgery. A systematic review of physical demands during sexual activity identified that most sexual positions for female partners involve flexion, abduction, and external rotation, whereas positions for male partners involve mainly external rotation [33]. For patients with femoroacetabular impingement, sexual positions that provoke impingement, particularly positions that involve combined hip flexion and abduction, were generally avoided [40]. It is common for patients to change sexual activity positions after an acetabular osteotomy or hip arthroscopic surgery [24, 46].

Two previous studies investigated specific sexual positions that were associated with pain during sexual activity before and after hip arthroscopy to correct femoroacetabular impingement [30, 43]. One study used computed tomography scans from ten patients with femoroacetabular impingement with cam morphology to develop digital models of hip impingement during sexual activity before and after cam correction [43]. Although no models of sexual positions were impingement‐free before surgery, 11 out of 29 positions were impingement‐free after surgery [43]. Positions involving a greater degree of hip flexion tended to have more impingement before cam correction, even at angles with flexion less than 90° [43]. For subsets of both male and female partners, there was an overall significant decrease in impingement throughout all positions [43]. A second study with computational modelling investigated hip instability and impingement in common sexual positions after hip arthroscopy to correct femoroacetabular impingement [30]. Instability was noted in 67.8% of positions for male partners (largely due to excessive external rotation) and 35.7% of positions for female partners (due to excessive abduction) [30]. Impingement was observed in 33.3% of male positions (due to excessive adduction) and 42.9% of female positions (due to excessive flexion or internal rotation) [30]. Overall, patients should be counselled that there is a risk of hip instability after hip arthroscopy, therefore certain sexual positions should be avoided [30]. Patients should be aware that positions for sexual activity may change after hip surgery for hip dysplasia or femoroacetabular impingement.

Although most studies agree that sexual function is improved after hip sports medicine orthopaedic surgeries, it is important that patients are counselled on the risk of iatrogenic nerve injuries during surgery. The use of a perineal post during hip arthroscopy is implicated in almost a quarter of all intraoperative complications with this surgery [13]. Importantly, the pudendal nerve is at risk for injury in hip arthroscopy. The pudendal nerve is a major nerve responsible for sexual function [3]. In female patients, the pudendal nerve may be implicated in symptoms of vulvodynia associated with femoroacetabular impingement [9]. For male patients, damage of the pudendal nerve can contribute to erectile dysfunction after surgery. In patients with ongoing pudendal nerve damage or entrapment, potential therapeutic measures include anaesthesia injection, decompression or neurolysis [3]. To avoid pudendal nerve damage intra‐operatively, one study in this review conducted hip arthroscopy without a perineal post [49]. The authors found that in a postless hip arthroscopy, 0% of patients had iatrogenic nerve injuries [49]. Further studies comparing hip arthroscopy with and without the use of a perineal post could be conducted to verify the benefit of postless hip arthroscopy on reducing pudendal nerve injury. Other major nerves implicated in iatrogenic nerve injuries were the sciatic nerve and lateral femoral cutaneous nerve. In a systematic review, deep gluteal syndrome causing sciatic nerve entrapment was iatrogenic in 30% of cases [19]. Similarly to the pudendal nerve, damage to the sciatic nerve is likely due to traction. In contrast, damage to the lateral femoral cutaneous nerve is likely a consequence of portal placement [32]. Most nerve injuries reported in the current review were temporary and resolved spontaneously. However, patients should be counselled about the risk of permanent nerve damage.

Patient‐reported outcome measures (PROMs) are an important tool for assessing the severity of a patient's condition and how it affects their life; however, sexual function is rarely included in these assessments. Of commonly used PROMs for hip arthroscopy, only the iHOT‐12 specifically asks “how much trouble do you have with sexual activity because of your hip?”. In this review, only two papers involving hip procedures included this PROM [7, 42]. By not including questions about sexual function in commonly used PROMs, there is a missed opportunity to collect data about this vital part of holistic patient wellness. Future research should be directed towards modifying existing PROMs to capture sexual function.

There are some limitations of this review. First, all but one of the studies included in the current review were level of evidence IV. This one study was level of evidence III [6]. This reflects the fact that most of the studies in the current review were case series or cohort analyses. Additionally, the heterogeneity of the results extracted from the included references precluded the ability to conduct a pooled analysis of the results. Many studies reported on qualitative measures of sexual function, such as pain during sexual activity or self‐perceived sexual function. There it was difficult for us to present pooled outcomes and analysis on validated scores, such as the SAQ, IIEF, IIEF‐5 and the FSFI. Most studies solely considered heterosexual sexual encounters with cisgender patients, which limits the generalisability of the results to LGBTQIA+ populations. Lastly, due to the sensitive nature of the current topic, it is possible that patients under‐reported sexual difficulties. There may be a selection bias in patients who agreed to answer questionnaires or report on their sexual activity.

## CONCLUSION

Hip sports medicine surgeries can improve sexual function for patients; however, they have a risk of pudendal nerve damage that can temporarily interfere with sexual function. Surgeons should counsel their patients on the risks and benefits of hip sports orthopaedic surgeries to sexual function.

## AUTHOR CONTRIBUTIONS

**Madeline Hubbard**: Screening; extraction; writing. **Darya Pascarel**: Screening; extraction; writing. **Prushoth Vivekanantha**: Writing; editing; supervision. **Mahmoud Almasri**: Writing; editing. **Shahbaz Malik**: Writing; editing. **Amit Meena**: Writing; editing. **Darren de SA**: Writing; editing; supervision.

## CONFLICT OF INTEREST STATEMENT

The authors declare no conflicts of interest.

## ETHICS STATEMENT

There are no relevant ethical disclosures pertaining to research involving human participants and/or animals, and informed consent was not necessary to develop this manuscript.

## Supporting information

Supporting information.

## Data Availability

Data may be made available upon reasonable request at prushoth.vivekanantha@medportal.ca.
