# Supplementary material for: Hip arthroscopy and periacetabular osteotomy generally improve sexual function in patients, but have a risk of iatrogenic pudendal nerve injury that can temporarily worsen sexual function: A systematic review
Source: Knee Surg Sports Traumatol Arthrosc. 2025 May 19;33(7):2642–54. doi: 10.1002/ksa.12700 (PMC12205418; doi:10.1002/ksa.12700)
Supplement: Supplementary file 1 — Supporting information. [file KSA-33-2642-s001.docx]

**SUPPLEMENTARY DIGITAL MATERIAL:**

**Supplementary Table 1. Search Criteria**

| **PubMed (n=2000)** | **EMBASE (n=2563)** | **MEDLINE (n=1219)** |
| --- | --- | --- |
| 1. Sexual OR coit* | 1. Sexual OR coit* | 1. Sexual OR coit* |
| 1. Health OR education OR intercourse OR activity OR function OR satisfaction | 1. Health OR education OR intercourse OR activity OR function OR satisfaction | 1. Health OR education OR intercourse OR activity OR function OR satisfaction |
| 1. orthopaedic OR orthopaedic OR fracture OR ankle OR hip OR knee OR shoulder | 1. orthopaedic OR orthopaedic OR fracture OR ankle OR hip OR knee OR shoulder | 1. orthopaedic OR orthopaedic OR fracture OR ankle OR hip OR knee OR shoulder |
| 1 AND 2 AND 3 | 1 AND 2 AND 3 | 1 AND 2 AND 3 |
